# Synthetic Messenger RNA-Based Vaccines: From Scorn to Hype

**DOI:** 10.3390/v13020270

**Published:** 2021-02-09

**Authors:** Steve Pascolo

**Affiliations:** 1 Department of Dermatology, University Hospital of Zürich, Gloriastrasse 31, 8091 Zürich, Switzerland; steve.pascolo@usz.ch; Tel.: +41-4463-42877; 2 Faculty of Medicine, University of Zürich, 8091 Zürich, Switzerland

**Keywords:** mRNA, SARS-CoV-2, vaccine, spike protein

## Abstract

In the race for a vaccine against SARS-CoV-2, the synthetic mRNA format has been shown to be the fastest one and proved to be safe and highly efficient, even at the very low dose of a few µg per injection. The mRNA vaccines are not new: vaccines that are based on attenuated mRNA viruses, such as Mumps, Measles, and Rubella, immunize by delivering their mRNAs into the cells of the vaccinated individual, who produces the viral proteins that then prime the immune response. Synthetic mRNA in liposomes can be seen as a modern, more refined, and thereby a safer version of those live attenuated RNA viruses. The anti-COVID-19 mRNA vaccine (coding the SARS-CoV-2 spike protein) is the third synthetic RNA therapeutic being approved. It follows the aptamer Macugen^®^ (which neutralizes VEGF) and the siRNA Onpattro^®^ (which destroys the transthyretin-coding mRNA). Remarkably, the 30 µg of mRNA that are contained in the first approved anti-COVID-19 vaccine are sufficient for generating high levels of neutralizing antibodies against the virus in all injected volunteers (including participants over 65 years old). The efficacy and safety data are stunning. The distribution of these vaccines throughout the world will bring a halt to the coronavirus pandemic.

## 1. Introduction

**Figure 1:**
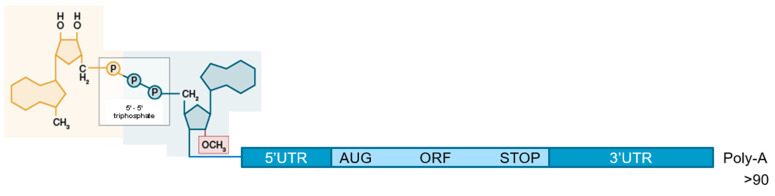
The structure of the in vitro transcribed (ivt) mRNA. The mRNA starts with a cap structure, followed by an untranslated region (5′ UTR) up to the start codon. Between the start and stop codon is the coding sequence. This is followed by the 3′ UTR and a poly-A tail.

RNA is the only biological molecule that can recapitulate, on its own, all of the characteristic functions of life (including storage, regulation, and replication of genetic information in organized three-dimensional (3D) structures) [1] and several of these functions are being exploited to generate therapeutics (see Pascolo, S. in Drug Discovery Handbook (ed Shayne Cox Gad) Ch. 27, (2005)):

- RNA contains genetic information and, thereby, is being used to allow for the organism to encode a specific protein of interest (Figure 1); this approach has been optimized for nearly 30 years to be used as a vaccine platform [2,3,4,5] and just reached approval in December 2020 (with the first vaccine against COVID-19);
- RNA forming 3D structures, as in ribosomes or any ribo-nucleoprotein complexes, is used to generate aptamers. This led to the design of Macugen^®^ (a structured 2′ Fluoro oligonucleotide, which binds and blocks VEGF) for the treatment of Age-Related Macular Degeneration;
- RNA can perform enzymatic activities as evidenced for ribozymes (this technology has not yet been turned into an approved drug);
- RNAs control gene expression, as seen with micro RNAs (miRNAs) that has been turned into a drug (siRNA) that degrades messenger RNA encoding transthyretin and it is used for the treatment of hereditary transthyretin amyloidosis.

Interestingly, the newcomer in this list of synthetic RNA therapeutics, the vaccine against SARS-CoV-2 [6], requires remarkably low doses: 30 µg of synthetic in vitro transcribed mRNA (ivt mRNA) that is injected twice into the muscle, while Macugen^®^ (OSI Pharmaceuticals, Melville, NY, USA) is given intravitreal at a dose of 3000 µg every six weeks and Onpattro^®^ (Alnylam Pharmaceuticals, Inc., Cambridge, MA, USA) is intravenously delivered at a dose up to 30,000 µg every three weeks. This very low dose of the mRNA vaccine as well as its safety and efficacy features make it the superlative vaccine that is needed to curb the COVID-19 pandemic. It has been approved for emergency use in the UK (2 December 2020) and in the US (11 December 2020), but also under regular authorization conditions in Switzerland (19 December 2020) and the EU (21 December 2020).

## 2. History of Synthetic Non-Replicating mRNA Vaccines

It can be considered that the vaccines against yellow fever, measles, mumps, and rubella are early versions of mRNA vaccines: These attenuated viruses are injected subcutaneously and without adjuvants to deliver their RNA genomes into the cells of the host. The infected cells produce the viral proteins and sense danger, which leads to the triggering of inflammation, allowing for the development of an immune response. The exact same mechanisms account for the functionality of the ivt mRNA vaccines. However, instead of a viral membrane containing many components (lipids and proteins), the ivt mRNA vaccine envelope that is used in the current anti-COVID-19 vaccines is made of a liposome consisting of a few (usually four) defined lipids. In addition, instead of complex mRNAs coding for many viral proteins, the ivt mRNA vaccine contains a single mRNA coding for one (in the case of non-replicating mRNA vaccines) selected protein: a structural protein from an infectious agent, an allergen, or a tumor antigen, for example. Although ivt mRNA vaccines can come in two flavors: replicating (encoding an antigen and an RNA replicase that amplifies the recombinant mRNA) and non-replicating (encoding only the antigen), this article will focus on non-replicating ivt mRNA vaccine as it is this format that is utilized and approved in the vaccines combating COVID-19 [6].

The possibility to use non-replicating ivt mRNA to generate vaccines was first disclosed by Martinon and colleagues in 1993 [7]: Ivt mRNA coding for the influenza virus nucleoprotein (NP) and encapsulated into cholesterol/phosphatidylcholine/phosphatidylserine liposomes induced NP-specific cytotoxic T-cells in mice that had received the vaccine (12 µg as a prime-boost regimen) sub-cutaneous or intravenously (intraperitoneal injections were inefficient). Two years later, Conry et al. [8] published that ivt mRNA coding Carcino Embryonic Antigen (CEA) and injected naked (just in saline, without any cationic carrier or liposome) in the muscle can prime an antibody response against CEA that becomes evident after challenge of the mice with CEA-expressing tumor cells (injections were twice weekly for five weeks with 50 µg of ivt RNA encoding CEA), i.e., a total amount of 500 µg of mRNA per mouse. Subsequently, in 2000, Hoerr and colleagues [9] published that a single intradermal injection of 30 µg of mRNA either naked (in saline) or protected by the cationic peptide Protamine, as well as intravenous injection of mRNA in a liposome, induced an immune response against the encoded protein (the model antigen β-galactosidase). It should be noted that all those three pioneering studies have been published in scientific journals of moderate impact factors. The same is true of the first reports of injection in a human participant (actually in myself!) [10] and the first human clinical trials (see below) [11,12,13]. At this early stage, the scientific and medical communities did not see the potential of this vaccination strategy. As opposed to Martinon et al. and Conry et al., Hoerr et al. turned their pre-clinical work into further development and clinical studies by creating a dedicated company: CureVac in 2000. Although I am co-founder of the company and was the chief scientific officer from 2000 until 2006, I left the company in 2006 and have, since then, no collaborations with the company as well as no shares. Fortunately, in the early 00s, investors were more open to this technology than the scientific community and the company obtained private investments (from business angels and then risk capital). Some public money has also been available to us from an EU consortium on vaccination against Classical Swine Fever Virus (EU grant QLK2-CT-2001-01346) [14] and through the support of the University of Tuebingen (via the “Jung Innovatoren Program”). From this, we could purchase some basic laboratory equipment for a start-up laboratory that was provided by the University of Tuebingen as part of the “Jung Innovatoren” before moving in 2003 into the newly built technology park of the city of Tuebingen. As a co-founder and Chief Scientific Officer, my tasks where mainly to (i) improve the mRNA vaccine (i.e., the structure and delivery of the mRNA), (ii) establish a pharmaceutical production in-house, and (iii) implement the first clinical studies in humans.

The first task was achieved by optimizing the mRNA (by the design and the purification by an original chromatography purification method of mRNA according to size [2]), delivery solution of mRNA for intradermal injection [10], testing adjuvants to the vaccine [15], and understanding the immunostimulatory capacities of RNA [16,17].

The second task, which was particularly supported by the local public authorities, was constructed in the newly built technology park, a GMP suite that we designed for our process of mRNA production from plasmid DNA purification in class D rooms, up to mRNA production and purification by HPLC [2,10] in class C rooms and final packaging in class A cabinets. The validation of the process, as well as in-process and end-product control, was a constructive task, since molecular biology methods, although well-established, had to be particularly clearly defined and demonstrated to be robust and accurate. The company obtained the manufacturing authorization in 2006. The GMP-quality mRNA was found to be better than laboratory grade mRNA (probably because of the chromatography purification step that we were the first to use for therapeutic mRNA) [10]. I was intradermally injected with ivt mRNA coding for luciferase that was produced in those conditions. Firefly luciferase activity was evidenced in punch biopsies made in my skin at the sites of injection a few hours after administrations [10]. This demonstrated that, similar to mouse skin, human skin can take up injected ivt mRNA formulated in an isotonic buffer.

The third task was facilitated by several specific aspects of the German law on medical products that, for example, allowed a physician to produce medicinal products and use them personally (as long as this was done under the direct professional responsibility without this production of medicinal products being covered by the provisions of the Medicinal Products Act). As early as 2003, we administered ivt mRNA vaccines intradermally into cancer patients. This first study was a personalized vaccine, where mRNA was extracted from tumor biopsies, reverse transcribed in cDNA libraries, transcribed into ivt mRNA [18], formulated, and injected in the very same patient from whom the biopsy originated [12]. This study, which was published in 2008, demonstrated the safety of the approach and indicated that it could induce an increase in the anti-tumor humoral immune response. It was followed by two other trials: one in melanoma patients published in 2009 [13] and one in renal cell carcinoma patients published in 2010 [11]. In both cases, an adapted mixture of six defined ivt mRNAs, coding adequate tumor antigens, was intradermally injected: naked for the renal cell carcinoma patients and complexed with Protamine for the melanoma patients. Both of the studies have indicated that the ivt mRNA vaccine formulations are safe, induce immune responses, and some clinical responses were reported [11,13,19]. 

Despite these promising results, a phase IIb trial evaluating the efficacy of an ivt mRNA vaccine coding PSA, PSCA, PSMA, and STEAP1 as a monotherapy against prostate cancer failed to show efficacy (as assessed by overall survival and progression-free survival) of the approach. However, the failure was not from the vaccine itself (an immune response against proteins encoded by the ivt mRNAs was detected) [20], but from the immune response itself: it could not control tumor growth enough to result in a favorable clinical outcome. Several trials using ivt mRNA as anti-cancer vaccines are ongoing and the design of the ivt mRNA (e.g., coding for mutations), the formulation (e.g., in liposomes given intravenous) as well as the regimen for its utilization (e.g., in combination with other cancer treatments) will surely lead to the validation of anti-cancer ivt mRNA vaccines [21,22]. Meanwhile, ivt mRNA vaccines have been evaluated in early human clinical studies as prophylactic vaccines against viruses: including influenza (with a dose range between 25 and 400 µg intramuscular or intradermal) [23] and rabies (with a dose range between 80 and 640 µg via intramuscular or intradermal administration) [24]. Other phase I/II clinical studies of ivt mRNA vaccines against several other viruses (Cytomegalovirus, Zika virus, Metapneumovirus, and Parainfluenza Virus Type 3) are currently ongoing. Most importantly, the anti-SARS-CoV-2 vaccine in the form of an ivt mRNA was recently approved in several countries and from two independent companies in a dose range of 30 to 100 µg, as given by intramuscular injection.

## 3. Advantages of Synthetic mRNA Vaccines

**Figure 2:**
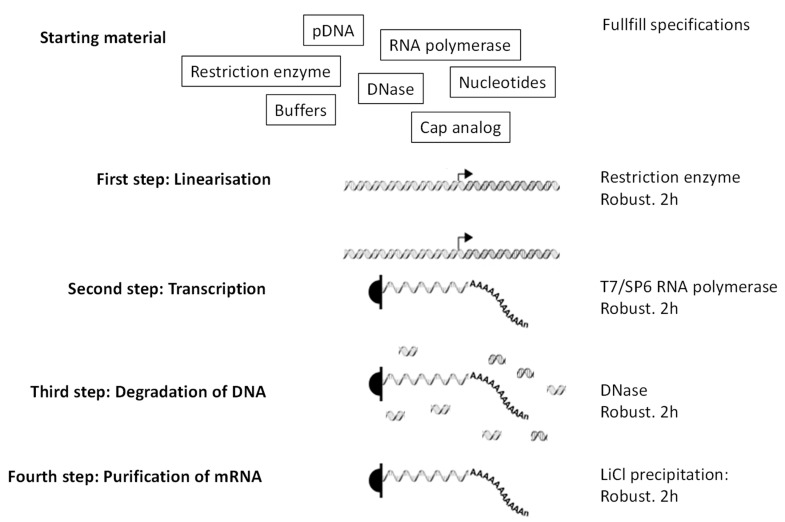
Production of ivt mRNA. Little starting material is needed. Then the four steps are all robust (work independently of the sequence of the ivt mNA needed) and take only a few hours each (each step takes 2 h in the lab). Right arrow is a promoter recognized specifically by the RNA polymerase.

Although, in the laboratory, the production and utilization of ivt mRNA for therapies are cumbersome and expensive, it appears that, for clinical applications, this format has on the contrary many advantages compared to other approaches:

- Fast and easy production in pharmaceutical conditions. The steps leading to production of mRNA (Figure 2) are simple, fast, and most importantly robust, which means that they do not depend on the mRNA sequence. Thus, once the pharmaceutical process for mRNA production is established, it can be used for any RNA sequence coding any protein. That is not the case for vaccines that are based on peptides, proteins, nanoparticles, or viruses. In those cases, preexisting knowledge and infrastructures do not guarantee, in the context of a new pathogen/antigen, that production will be fast and successful. Peptides, depending on their sequences, can be very difficult to synthesize, to purify, or to store. Proteins, depending on their structure and glycosylation status, must be expressed by adequate organisms (to be individually defined) and their purification and preservation is a specific project for each new protein. Similarly, production, purification, and preservation of attenuated or inactivated viruses require extensive testing and optimization to define the methods that are adapted to the precise pathogen of interest. The versatility of mRNA production is key to producing individualized anti-cancer vaccines: tumor biopsies are sequenced, cancer-specific mutations identified, and ivt mRNA are produced in order to encode short proteins that contain identified mutations (http://merit-consortium.eu/about/ (accessed on 06 February 2021)). Each patient receives a personalized vaccine. The whole process, from the biopsy to the tailor-made mRNA vaccine, takes less than three months. Thus, mRNA companies are accustomed to producing ivt mRNA vaccines from whatever nucleotide sequence within less than three months.
- Highly stable in vitro. Contrary to the general belief, RNA is very stable in vitro. It is degraded in the presence of RNases or at elevated pH. However, should the RNA be free of RNases (which is of course the case for a pharmaceutical ivt mRNA) and at neutral/slightly acidic pH, it is then extremely stable. It is the only biological molecule that can be precipitated, resuspended in water, heated up to 90 degrees Celsius, frozen, and lyophilized, all without damage. No other biological product is as robust (particularly concerning the elevated temperatures). In addition, lyophilized RNA can be stored at room temperature for a very long time. Being resuspended in water, it will again be immediately biologically active (independently of its sequence, i.e., independently of the encoded protein). On this basis, in 2014 CureVac received a special prize of two million Euros from the EU (https://ec.europa.eu/commission/presscorner/detail/en/IP_14_229 (accessed on 06 February 2021)). The prize was for innovative solutions for vaccine transportation and storage where the cold chain cannot be guaranteed.
- Safe and defined therapeutic window. As opposed to DNA-based vaccines (i.e., plasmid DNA or DNA viruses), the ivt mRNA is, per nature, a transient molecule in vivo. It is totally degraded by abundant RNases outside and inside cells. It can also not affect the DNA genome of the host. For those reasons, ivt mRNA is particularly safe for therapies. There is another advantage of the very transient nature of ivt mRNA in vivo: after a time that can be evaluated, to a maximum of a few days to less than a week (depending on the mRNA sequence and delivery vehicle), it can be assumed that the ivt mRNA is no longer active. Thus, the therapeutic window is very well defined. Re-injections can be performed and they will not cumulate with previous injections. Therefore, there can not appear any surprising side effects that are associated with a cumulative aspect and individuals can be repeatedly injected, as required, for the efficacy of the treatment.
- Mono-antigenic. Vaccines in the form of recombinant viruses express many different proteins, including the one of interest (SARS-CoV-2 Spike protein, for example). Thus, boost vaccines are additionally (i) triggering immunity against non-targeted antigens and can (ii) be neutralized by antibodies triggered by the prime against the vehicle. A similar problem can be seen with complex recombinant vaccines (virus-like particles) or even protein vaccines that, by nature, contain contaminants (e.g., degradation products, misfolded proteins, or impurities from production) that may trigger and boost immune responses that are not the ones of interest.

## 4. Design and Optimization of Non-Replicating Ivt mRNAs

Although it is a simple formulation, the ivt mRNA vaccine can be optimized in many ways. This optimization has started over 20 years ago and several very important achievements can be reported on both the mRNA itself and the delivery method.

- Optimizing the mRNA. Four functional parts in the molecule can be defined and each can be independently optimized in order to lead to superlative mRNA molecules that are (i) more stable in the cytosol of cells and (ii) better translated, as shown in Figure 1. It should be noted that these two characteristics are correlated and that usually the most stable mRNAs are also best translated and vice-versa.

Let us describe those elements and their optimization from the 5′ to the 3′ end. At the 5′ end is the “cap” structure. It is recognized by Eukaryotic translation initiation factor (eIF4E of the eIF4F complex), which then builds the translation machinery around the mRNA. The 5′ cap consists of a guanosine that is methylated on position 7 and linked with a tri-phosphate bond to the first nucleotide of the RNA. Ideally, this first nucleotide is methylated on position 2′. That gives the “cap1” structure. For many years a cap analog was used for in vitro transcription, whereby the RNA polymerase was starting mRNA synthesis using a G residue and the transcription reaction would contain a five-fold excess of cap analog m 7 G (5′) ppp (5′) G or, even better, an anti-reverse cap analog 3′-O-Me-m 7 G (5′) ppp (5′) G. This would allow for statistically 80% of the synthesized mRNA to start with a cap and 20% to start with a G. The mRNAs starting with a G are not translated, as they cannot be recognized by the eIF4F complex. Not only did this method give a proportion of uncapped mRNA, it also made the transcription reaction less efficacious: G residues were in five-fold lower concentrations than other residues and they became the limiting parameter during the transcription reaction. This method usually yielded one milligram of mRNA per milliliter of transcription. A revolution came from Trilink in 2017, using the CleanCap system (TriLink Biotechnologies, San Diego, CA, USA). It is a trinucleotide consisting of the 7-methyl guanosine that is linked by a triphosphate bond to a 2′ methylated adenosine followed by a guanosine. This trinucleotide is the only way for a polymerase to start transcription when the mRNA is starting by an A residue (the wild type promoter used for ARCA cap is starting mRNA at a G residue). CleanCap has two exceptional features: It allows incorporation of the cap1 at the 5′ end of virtually all of the synthesized mRNAs and it does not limit the concentration of any of the four nucleotides. In this way, the transcription reaction gives 5–10 milligrams of mRNA per milliliter of transcription. Nowadays, all ivt mRNA productions in the larger mRNA companies (see below) use CleanCap, as this strategy is, so far, incomparably better than those that are based on other cap analogs.

Moving to the next functional structure: the 5′ untranslated region (UTR), which is located between the cap and the start codon. It can be as short as three nucleotides [25] or containing hundreds of residues. The ribosome scans this UTR and starts translation at the first AUG codon in a correct surrounding sequence (called the Kozak sequence). The sequence of this 5′ UTR can regulate translation. For example, “upstream open reading frames (uORFs)” can control gene expression [26]. Usually, the 5′UTR of very stable mRNAs, such as those utilized by the globin-coding mRNA, are used in ivt mRNA. We described an optimized 5′UTR in the form of an aptamer capable of attracting the eIF4F complex [27]. Other optimized sequences were defined using predictions or iterative methods [28]. Companies use usually their own proprietary 5′UTR sequence.

Following the 5′UTR is the coding sequence. This part is crucial for the rate of mRNA translation and as a correlate the stability of mRNA in the cytosol. Although wild type sequences of mammalian genes are usually naturally adapted to our codon usage, sequences from lower organisms (for example, bacteria) can be inadequate for efficacious translation in a mammalian cytosol. Thus, codon optimization can be required or not. In any case, codon optimization potentially improves the translation of an mRNA. However, we experienced some failures that could be explained, for example, by the fact that a too rapid translation can result in misfolding of a protein. Generally, rare codons can be located at positions between folding domains, where translation must slow down, so that a protein domain can get properly structured before the rest of the protein is synthesized. Thus, codon optimization must be carefully applied and optimized for each ivt mRNA project. When the genes are from mammalian viruses (like SARS-CoV-2) and the time to test many different codon optimized sequence is lacking, the use of the wild type sequence can be recommended.

After the coding sequence, ending on one of the three stop codons (Amber, Ochre, or Opal) and before the poly-A tail, is the 3′UTR. It is a very critical sequence that is recognized by cytosolic protein as well as siRNA and it is very much impacting the mRNA stability. Here, again, early work used ivt mRNA having 3′UTRs from the very stable globin genes. More recently, tandem repeats of such sequences [29] or new sequences were defined [30]. As is the case for the 5′UTR, companies usually have their proprietary stabilizing 3′UTR in their ivt mRNAs and each provides efficacious intracellular stabilization to the synthetic molecule. The mRNA ends on a poly-A tail that ideally has over 90 residues. It can be contained in the DNA that is used for transcription or added on the ivt mRNA by poly-adenylation using a template-independent poly-A polymerase. The first method is preferrable during pharmaceutical production. The overall mRNA sequence can consist of the four canonical bases or in modified residues, such as pseudoUridine instead of uridine. This is not a critical feature in the context of ivt mRNA vaccines and companies either use or do not use modifications in their vaccine mRNAs (see below).

- Optimizing the vehicle. Cationic compounds can bind and stabilize the anionic RNA. At the same time, they neutralize the molecule and generate particles, facilitating uptake by cells and the passage through cell membranes. Cationic liposomes, as well as cationic polymers, have been used from the beginning of ivt mRNA vaccine optimization (liposomes and Protamines in the study by Hoerr [9]). Optimized formulations need to incorporate two opposing features: a very stable and resistant structure that protects RNA outside of the cells and a very efficacious dissociation of the RNA from its carrier inside the cytosol. Should the interaction between RNA and carrier be too strong, the RNA may not be released in the cytosol, and should it be too weak, the particles may be instable in vitro as well as after the injection and release RNA before it gets into the cytosol. In addition, the vehicle must consist of safe compounds that are catabolized naturally. Cationic liposomes (especially those containing ionizable cationic lipids) have shown, so far, to be the best. Their development has been accelerated, since the beginning of the century, for the delivery of siRNA drugs. These siRNAs must very efficiently reach the cytosol of cells to be efficacious. Companies, such as Acuitas Therapeutics, have made enormous progress in liposomal formulations aimed at delivering RNA, which facilitated the design of Onpattro^®^ (Alnylam Pharmaceuticals, Inc., Cambridge, MA, USA ), the first drug that is based on siRNA. As Onpattro^®^ (Alnylam Pharmaceuticals, Inc., Cambridge, MA, USA ) treats a genetic defect, it must be administered frequently and, as it controls gene expression in a large organ, it also needs to be given at a high dose. Thus, the formulation had to be efficacious and non-toxic. It consists of a delivery of up to 30 miligrams of RNA every three weeks by intravenous injection. Based on this expertise, safe and efficacious formulations of ivt mRNA could be developed. The liposome that is used to deliver the first approved ivt mRNA drug, the vaccine from BioNTech/Pfizer, is based on the technologies that were developed by Acuitas Therapeutics. CureVac is also using Acuitas Therapeutics formulations for its ivt mRNA vaccine against COVID-19 in clinical studies. Details on the formulations that were used by BioNTech, CureVac and Moderna for their anti-COVID-19 vaccines are given in a recent review by Buschmann et al. [31].

## 5. Anti-SARS-CoV-2 Non-Replicating mRNA Vaccines

From the start of the pandemic and solely based on the published sequence of the SARS-CoV-2 virus on 11 January in Nature, all three large mRNA companies: BioNTech (Mainz, Germany), CureVac (Tubingen, Germany), and Moderna (Boston, MA, USA) commenced to use their expertise and infrastructure to generate a vaccine against COVID-19. Fortunately, the SARS-CoV-2 virus from 2019 is similar to SARS-CoV from 2003 and, therefore, it could be rapidly determined that it also uses its spike protein to enter cells by recognizing ACE2, a membrane-bound enzyme that is expressed at the surface of many cells. The spike protein is a large (1273 amino acid residues in length) trans-membrane protein, with many glycosylations and fluctuating structures, which dictate its functions. It assembles as a trimer. From previous work on coronaviruses, it was known that the spike on virus particles is in its “pre-fusion” conformation. It is this form that must be recognized by antibodies aiming at neutralizing the virus. Two consecutive prolines in the C-terminal part of the spike replace the natural residues (sequence KV at amino acid position 986 and 987) in order to stabilize this conformation [32]. All three companies used this pre-existing knowledge to design their coding sequence: it includes the two prolines, favoring the pre-fusion structure of SARS-CoV-2 spike expressed from the ivt mRNA at the surface of the transfected cells. However, there are subtle differences between the three vaccines:

- Moderna: injecting the first volunteer on 16 March 2020, Moderna was the first drug developer to start clinical testing of an anti-COVID-19 vaccine. The single vaccine consists of a PseudoUridine-modified mRNA coding for the full-length pre-fusion conformation spike in a liposomal shell, and it was produced in record speed. The company has published the results of its phase I dose escalation study in November 2020 [33]. The three tested doses (in three cohorts of 15 participants) of 25, 100, and 250 µg ivt mRNA formulated in liposomes and given intramuscular as a prime-boost regimen (with four weeks interval between injections), induced high neutralizing antibody titers (similar or higher than the titers found in the sera from patients having recovered from COVID-19). In general, there have been side effects of the vaccine in half of the participants, such as fatigue, chills, headache, myalgia, and pain at the injection site. These side effects were more pronounced after the boost injection and at the highest dose of 250 µg. Clinical laboratory values revealed no severe issues. The company selected the middle dose of 100 µg for further studies and completed the enrollment of 30,000 participants for its phase III study on 22 October 2020. A preliminary analysis by the company indicated a 95% efficacy of the vaccine in protecting against COVID-19: 196 COVID cases (185 in placebo), 30 severe (30 placebo). Meanwhile, studies in vaccinated and virus-challenged non-human primates demonstrated that the vaccine could prevent virus replication in the upper and lower airways [34]. Based on the data that were provided by the company, the US FDA approved the vaccine for emergency use. This is the second approval (after the BioNTech vaccine, see below) of an mRNA drug, and the second approved vaccine against SARS-CoV-2.
- BioNTech: having a broad range of ivt mRNA technologies and wanting to provide the safest and most efficient vaccine against COVID-19, in January 2020 the company designed four different ivt mRNA vaccines: two mRNAs encoding the full-length pre-fusion conformation spike, one having pseudoUridines, and the other having unmodified Uridines (as there is currently no available evidence that, for this type of vaccine, whether one or the other version is advantageous), one mRNA coding for only the Receptor Binding Domain of the spike (as antibodies directed solely against this domain may be more potent and might avoid facilitated infection) and one corresponding to a self-amplifying mRNA (as this format requires much lower doses than non-replicating mRNA). Having started the first injection on 23 April 2020, BioNTech decided to move the pseudoU ivt mRNA coding for the full length pre-fusion conformation spike (BNT162b2) to its phase III study. The phase I data for this vaccine tested at doses of 10, 30, and 100 µg demonstrated seroconversion in all volunteers and high neutralizing antibody titers (again similar or higher than titers in the sera of patients having recovered from COVID-19), even in participants that were older than 65 years of age and, additionally, the vaccine showed a good safety profile [6]. Having announced their collaboration on 17 March 2020, Pfizer and BioNTech evaluated the vaccine together, strongly accelerating the recruitment of participants. The phase III study was completed as early as 18 November 2020. It involved over 40,000 participants and demonstrated 95% protection against COVID-19: 9 cases of COVID-19 at least seven days after the second dose were observed among vaccine recipients and 169 among placebo recipients. Related adverse events were observed for 21% of the vaccine recipients and 5% in the placebo group. Four vaccine-related serious adverse events were reported among vaccine recipients (shoulder injury that is related to vaccine administration, right axillary lymphadenopathy, paroxysmal ventricular arrhythmia, and right leg paresthesia). Severe fatigue was observed in approximately 4% of BNT162b2 recipients. Elevated temperatures (38.9 to 40 °C) were reported in 0.8% (vaccine) and 0.1% (placebo) recipients after the second dose. Based on these favorable safety and efficacy data, BNT162b2 became the first ever approved mRNA-based drug and first authorized vaccine against COVID-19 when the UK approved its implementation on 2 December 2020. Subsequently, the US have approved the vaccine under emergency use on 11 December 2020. On 19 December 2020, Switzerland granted a conditional marketing authorization to the BioNTech/Pfizer anti-SARS-CoV-2 mRNA vaccine that will be distributed under the name COMIRNATY. It is the first authorization worldwide that is not under emergency use.
- CureVac: having started its phase I study in June 2020, the company uses a liposome (cholesterol, 1,2-distearoyl-sn-glycero-3-phosphocholine (DSPC), PEG-ylated lipid, and a cationic lipid) intramuscular delivery of an unmodified mRNA coding for the full-length Spike in its pre-fusion conformation. The dose escalation went from 2 µg up to 12 µg. It is a prime boost regimen with 28 days between injections. The study population was 248 adults that were aged 18 to 60 years old. The company communicated (although the corresponding article is not yet published in a peer reviewed journal https://www.medrxiv.org/content/10.1101/2020.11.09.20228551v1.full-text (accessed on 06 February 2021)) that the vaccine was well tolerated (although the frequency and severity of adverse events increased with the dose level) and induced neutralizing antibodies in all subjects at the dose of 12 µg. The vaccine could also boost pre-existing immune responses in people who have been previously infected by SARS-CoV-2. At the end of September 2020, CureVac’s phase II study started and it included older adult participants. On 14 December 2020, the company announced the start of its pivotal clinical study.

## 6. Production and Storage

All three major players had pre-existing manufacturing facilities to produce ivt mRNA for injection. However, the mass production in a short time of a prophylactic vaccine is currently facing challenges. Ultimately, if one billion of doses are urgently required for each company (to vaccinate 500 million people twice, covering approx. 10% of the population: risk persons and the medical professional for example) it corresponds to 100 kg mRNA for Moderna, 30 kg mRNA for BioNTech and 12 kg ivt mRNA for CureVac. The volumes of in vitro transcriptions are then at least 20,000, 6000, and 2400 L, respectively (assuming a concentration of at least 5 mg/mL of mRNA at the end of the transcription reaction). One of the major issues here would not be only the transcription reaction or purification steps, but also the availability of the raw products: Mostly, CleanCap, triphosphate nucleotides, and enzymes. Those components are not produced by the mRNA companies themselves. They are purchased from specialized providers of biological reagents, such as Trilink and New England Biolabs, for example. Those providers had to upgrade their production capacities in order to deliver the required raw materials to mRNA producers. All of those chains of providers and manufacturers are being upscaled and the billions of doses will be available in 2021. For the year 2020, BioNTech/Pfizer has 50 million doses and Moderna 20 million doses. This has allowed the beginning of the vaccination campaigns in countries where the vaccine has been approved for use.

ivt mRNA, as a pure molecule (free of RNases), is very stable at room temperature, as mentioned above. However, the liposomal formulation may be unstable at room temperature and it may change in shape, size, or percentage of encapsulated mRNA, etc., by rounds of thawing/freezing. Thus, the distribution of the vaccine using adequate storage temperatures is a crucial prerequisite. So far, the BioNTech/Pfizer vaccine is shipped at −70 °C and it can be stored at this temperature for months and, once thawed, it can be stored for five days at 2–8 °C. The Moderna vaccine is shipped at −20 °C and it is stable at this temperature for months and is also expected to be stable at 2–8 °C for 30 days once initially thawed. CureVac has announced its vaccine remains stable for at least three months when stored at a standard refrigerator temperature of +5 °C and up to 24 h as a ready-to-use vaccine when stored at room temperature. It can be expected that all three companies will further provide stability data that will continuously allow easier transportation and distribution of the vaccines.

## 7. Conclusions

The scientific and medical communities regarded initiating an industrial development of an ivt mRNA vaccine in 2000 as utopist or irrelevant. Our pioneering work was rejected by grant evaluating panels and editors or reviewers of high impact journals. The first ever report of an ivt mRNA injection in human (being done in myself) is in Gene Therapy, impact factor 4, in 2007 (6). The first clinical studies of ivt mRNA vaccines were published in the Journal of Immunotherapy in 2008 and 2009, which also has an impact factor of 4 (8, 9). Now, through the COVID-19 pandemic, ivt mRNA vaccines have proven to be the fastest, safest, and very efficacious vaccine format. Enormous improvements of the method (ivt mRNA and carrier) has turned the utilization of a very small dose of ivt mRNA (30 µg per injection for the first approved vaccine, less than what was injected into mice 20 years ago) into a very potent vaccine. It is the first time in history that a vaccine has been validated within less than one year from the description of a new virus (the sequence of SARS-CoV-2 was released in January 2020). The ivt mRNA vaccines will participate in rescuing the world from the COVID-19 pandemic and it will solve many new future issues that arise from various infectious agents. In addition, ivt mRNA-based therapies can be used to address virtually any medical issue. Thus, this technology went from scorn to hype within 20 years and will now, in full, deliver in all its many promises.

## Data Availability

Not applicable.

## References

[1] Gilbert W. (1986). Origin of life: The RNA world. Nature.

[2] Pascolo S. (2004). Messenger RNA-based vaccines. Expert Opin. Biol. Ther..

[3] Pascolo S. (2006). Vaccination with messenger RNA. Methods Mol. Med..

[4] Pascolo S. (2008). Vaccination with messenger RNA (mRNA). Handb. Exp. Pharmacol..

[5] Sahin U., Kariko K., Tureci O. (2014). mRNA-based therapeutics—Developing a new class of drugs. Nat. Rev. Drug Discov..

[6] Polack F.P., Thomas S.J., Kitchin N., Absalon J., Gurtman A., Lockhart S., Perez J.L., Pérez Marc G., Moreira E.D., Zerbini C. (2020). Safety and Efficacy of the BNT162b2 mRNA Covid-19 Vaccine. N. Engl. J. Med..

[7] Martinon F., Krishnan S., Lenzen G., Magné R., Gomard E., Guillet J.G., Lévy J.P., Meulien P. (1993). Induction of virus-specific cytotoxic T lymphocytes in vivo by liposome-entrapped mRNA. Eur. J. Immunol..

[8] Conry R.M., LoBuglio A.F., Wright M., Sumerel L., Pike M.J., Johanning F., Benjamin R., Lu D., Curiel D.T. (1995). Characterization of a messenger RNA polynucleotide vaccine vector. Cancer Res..

[9] Hoerr I., Obst R., Rammensee H.G., Jung G. (2000). In vivo application of RNA leads to induction of specific cytotoxic T lymphocytes and antibodies. Eur. J. Immunol..

[10] Probst J., Weide B., Scheel B., Pichler B.J., Hoerr I., Rammensee H.G., Pascolo S. (2007). Spontaneous cellular uptake of exogenous messenger RNA in vivo is nucleic acid-specific, saturable and ion dependent. Gene Ther..

[11] Rittig S.M., Haentschel M., Weimer K.J., Heine A., Muller M.R., Brugger W., Horger M.S., Maksimovic O., Stenzl A., Hoerr I. (2011). Intradermal vaccinations with RNA coding for TAA generate CD8+ and CD4+ immune responses and induce clinical benefit in vaccinated patients. Mol. Ther..

[12] Weide B., Carralot J.P., Reese A., Scheel B., Eigentler T.K., Hoerr I., Rammensee H.G., Garbe C., Pascolo S. (2008). Results of the first phase I/II clinical vaccination trial with direct injection of mRNA. J. Immunother..

[13] Weide B., Pascolo S., Scheel B., Derhovanessian E., Pflugfelder A., Eigentler T.K., Pawelec G., Hoerr I., Rammensee H.G., Garbe C. (2009). Direct injection of protamine-protected mRNA: Results of a phase 1/2 vaccination trial in metastatic melanoma patients. J. Immunother..

[14] Ceppi M., de Bruin M.G., Seuberlich T., Balmelli C., Pascolo S., Ruggli N., Wienhold D., Tratschin J.D., McCullough K.C., Summerfield A. (2005). Identification of classical swine fever virus protein E2 as a target for cytotoxic T cells by using mRNA-transfected antigen-presenting cells. J. Gen. Virol..

[15] Carralot J.P., Probst J., Hoerr I., Scheel B., Teufel R., Jung G., Rammensee H.G., Pascolo S. (2004). Polarization of immunity induced by direct injection of naked sequence-stabilized mRNA vaccines. Cell. Mol. Life Sci..

[16] Scheel B., Braedel S., Probst J., Carralot J.P., Wagner H., Schild H., Jung G., Rammensee H.G., Pascolo S. (2004). Immunostimulating capacities of stabilized RNA molecules. Eur. J. Immunol..

[17] Scheel B., Teufel R., Probst J., Carralot J.P., Geginat J., Radsak M., Jarrossay D., Wagner H., Jung G., Rammensee H.G. (2005). Toll-like receptor-dependent activation of several human blood cell types by protamine-condensed mRNA. Eur. J. Immunol.

[18] Carralot J.P., Weide B., Schoor O., Probst J., Scheel B., Teufel R., Hoerr I., Garbe C., Rammensee H.G., Pascolo S. (2005). Production and characterization of amplified tumor-derived cRNA libraries to be used as vaccines against metastatic melanomas. Genet. Vaccines Ther..

[19] Rittig S.M., Haentschel M., Weimer K.J., Heine A., Müller M.R., Brugger W., Horger M.S., Maksimovic O., Stenzl A., Hoerr I. (2016). Long-term survival correlates with immunological responses in renal cell carcinoma patients treated with mRNA-based immunotherapy. Oncoimmunology.

[20] Kübler H., Scheel B., Gnad-Vogt U., Miller K., Schultze-Seemann W., Vom Dorp F., Parmiani G., Hampel C., Wedel S., Trojan L. (2015). Self-adjuvanted mRNA vaccination in advanced prostate cancer patients: A first-in-man phase I/IIa study. J. Immunothe.r Cancer.

[21] Kreiter S., Vormehr M., Van de Roemer N., Diken M., Löwer M., Diekmann J., Boegel S., Schrörs B., Vascotto F., Castle J.C. (2015). Mutant MHC class II epitopes drive therapeutic immune responses to cancer. Nature.

[22] Kranz L.M., Diken M., Haas H., Kreiter S., Loquai C., Reuter K.C., Meng M., Fritz D., Vascotto F., Hefesha H. (2016). Systemic RNA delivery to dendritic cells exploits antiviral defence for cancer immunotherapy. Nature.

[23] Feldman R.A., Fuhr R., Smolenov I., Ribeiro A.M., Panther L., Watson M., Senn J.J., Smith M., Almarsson Ö., Pujar H.S. (2019). mRNA vaccines against H10N8 and H7N9 influenza viruses of pandemic potential are immunogenic and well tolerated in healthy adults in phase 1 randomized clinical trials. Vaccine.

[24] Alberer M., Gnad-Vogt U., Hong H.S., Mehr K.T., Backert L., Finak G., Gottardo R., Bica M.A., Garofano A., Koch S.D. (2017). Safety and immunogenicity of a mRNA rabies vaccine in healthy adults: An open-label, non-randomised, prospective, first-in-human phase 1 clinical trial. Lancet.

[25] Elfakess R., Dikstein R. (2008). A translation initiation element specific to mRNAs with very short 5’UTR that also regulates transcription. PLoS ONE.

[26] Castelo-Szekely V., De Matos M., Tusup M., Pascolo S., Ule J., Gatfield D. (2019). Charting DENR-dependent translation reinitiation uncovers predictive uORF features and links to circadian timekeeping via Clock. Nucleic Acids Res..

[27] Tusup M., Kundig T., Pascolo S. (2018). An eIF4G-recruiting aptamer increases the functionality of in vitro transcribed mRNA. Int. J. Med. Health Sci..

[28] Sample P.J., Wang B., Reid D.W., Presnyak V., McFadyen I.J., Morris D.R., Seelig G. (2019). Human 5′ UTR design and variant effect prediction from a massively parallel translation assay. Nat. Biotechnol..

[29] Holtkamp S., Kreiter S., Selmi A., Simon P., Koslowski M., Huber C., Türeci O., Sahin U. (2006). Modification of antigen-encoding RNA increases stability, translational efficacy, and T-cell stimulatory capacity of dendritic cells. Blood.

[30] von Niessen A.G., Poleganov M.A., Rechner C., Plaschke A., Kranz L.M., Fesser S., Diken M., Löwer M., Vallazza B., Beissert T. (2018). Improving mRNA-Based Therapeutic Gene Delivery by Expression-Augmenting 3′ UTRs Identified by Cellular Library Screening. Mol. Ther..

[31] Buschmann M.D., Carrasco M.J., Alishetty S., Paige M., Alameh M.G., Weissman D. (2021). Nanomaterial Delivery Systems for mRNA Vaccines. Vaccines.

[32] Pallesen J., Wang N., Corbett K.S., Wrapp D., Kirchdoerfer R.N., Turner H.L., Cottrell C.A., Becker M.M., Wang L., Shi W. (2017). Immunogenicity and structures of a rationally designed prefusion MERS-CoV spike antigen. Proc. Natl. Acad. Sci. USA.

[33] Jackson L.A., Anderson E.J., Rouphael N.G., Roberts P.C., Makhene M., Coler R.N., McCullough M.P., Chappell J.D., Denison M.R., Stevens L.J. (2020). An mRNA Vaccine against SARS-CoV-2—Preliminary Report. N. Engl. J. Med..

[34] Corbett K.S., Flynn B., Foulds K.E., Francica J.R., Boyoglu-Barnum S., Werner A.P., Flach B., O’Connell S., Bock K.W., Minai M. (2020). Evaluation of the mRNA-1273 Vaccine against SARS-CoV-2 in Nonhuman Primates. N. Engl. J. Med..

